# 18q12.3‐q21.1 microdeletion detected in the prenatally alcohol‐exposed dizygotic twin with discordant fetal alcohol syndrome phenotype

**DOI:** 10.1002/mgg3.1192

**Published:** 2020-02-25

**Authors:** Hanna Kahila, Heidi Marjonen, Pauliina Auvinen, Kristiina Avela, Raili Riikonen, Nina Kaminen‐Ahola

**Affiliations:** ^1^ Department of Obstetrics and Gynecology Helsinki University Hospital and University of Helsinki Helsinki Finland; ^2^ Department of Medical and Clinical Genetics Medicum University of Helsinki Helsinki Finland; ^3^ Department of Clinical Genetics Helsinki University Hospital HUSLAB Helsinki Finland; ^4^ Children's Hospital Kuopio University Hospital University of Eastern Finland Kuopio Finland

**Keywords:** 18q deletion syndrome, comparative genomic hybridization array, DNA methylation, fetal alcohol spectrum disorders, fetal alcohol syndrome, growth retardation, *IGF2/H19*, prenatal alcohol exposure, twins

## Abstract

**Background:**

A pair of dizygotic twins discordantly affected by heavy prenatal alcohol exposure (PAE) was reported previously by Riikonen, suggesting the role of genetic risk or protective factors in the etiology of alcohol‐induced developmental disorders. Now, we have re‐examined these 25‐year‐old twins and explored genetic origin of the phenotypic discordancy reminiscent with fetal alcohol syndrome (FAS). Furthermore, we explored alterations in DNA methylation profile of imprinting control region at growth‐related insulin‐like growth factor 2 (*IGF2*)*/H19* locus in twins' white blood cells (WBC), which have been associated earlier with alcohol‐induced genotype‐specific changes in placental tissue.

**Methods:**

Microarray‐based comparative genomic hybridization (aCGH) was used to detect potential submicroscopic chromosomal abnormalities, and developmental as well as phenotypic information about twins were collected. Traditional bisulfite sequencing was used for DNA methylation analysis.

**Results:**

Microarray‐based comparative genomic hybridization revealed a microdeletion 18q12.3‐q21.1. in affected twin, residing in a known 18q deletion syndrome region. This syndrome has been associated with growth restriction, developmental delay or intellectual deficiency, and abnormal facial features in previous studies, and thus likely explains the phenotypic discordancy between the twins. We did not observe association between WBCs’ DNA methylation profile and PAE, but interestingly, a trend of decreased DNA methylation at the imprinting control region was seen in the twin with prenatal growth retardation at birth.

**Conclusions:**

The microdeletion emphasizes the importance of adequate chromosomal testing in examining the etiology of complex alcohol‐induced developmental disorders. Furthermore, the genotype‐specific decreased DNA methylation at the *IGF2/H19* locus cannot be considered as a biological mark for PAE in adult WBCs.

## Graphical abstract

Methylation profiles of imprinting control region at the IGF2/H19 locus in white blood cells of prenatally alcohol‐exposed adult twins and control women.

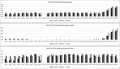

## 1 INTRODUCTION

Fetal alcohol spectrum disorders (FASDs) are a consequence of prenatal alcohol exposure (PAE), and an umbrella term for all alcohol‐related neurodevelopmental disorders and birth defects. The most severe form of FASD is fetal alcohol syndrome (FAS), with growth restriction, craniofacial dysmorphology, and central nervous system defects. In addition to timing, the amount and frequency of alcohol exposure also genetic background of fetus affects the vulnerability to the teratogenic effects of PAE (Eberhart & Parnell, 2016; Mead & Sarkar, 2014). Several twin studies have suggested that genetic factors, susceptibility or resistance alleles, could affect the severity of phenotype (Hemingway et al., 2018; Streissguth & Dehaene, 1993). However, any causality between a genetic factor, molecular mechanism, and the phenotype has not been identified so far. In our recent study, we observed a single‐nucleotide polymorphism (SNP) rs10732516 (NC_000011.10:g.1999976G > A) at the regulation region of insulin‐like growth factor 2 *(IGF2)* (OMIM *147,470)*/*H19 imprinted maternally expressed transcript *(H19)* (OMIM *103,280) locus which associates in parent‐of‐origin manner with altered placental DNA methylation and phenotype of alcohol‐exposed newborns (Marjonen, Kahila, & Kaminen‐Ahola, 2017). However, the functionality of this SNP has not been yet revealed.

We have re‐examined a discordant twin pair reported by Riikonen (1994) to reveal the genetic background of the phenotypic discordancy. The 26‐year‐old mother of the twins was a heavy consumer of alcohol, which was reported by personal interview (RR). The mother of four (P4) consumed 10 bottles of beer almost daily (120 g of pure alcohol) throughout the second half, but not during the first half of pregnancy. However, it is not clear when the pregnancy was recognized and alcohol consumption broken off. Twins were delivered by cesarean section at the 38th gestational week due to breech presentation of twin B. The pregnancy was otherwise unremarkable and the mother did not use drugs. The placenta was characterized by dichorionic twinning. Early characteristics of dizygotic twins can be seen in Table 1. Twin B fulfilled the criteria of FAS: prenatal growth retardation, slow psychomotor development as well as minor abnormalities, and stigmata of the face (Riikonen, 1994).

**Table 1:** Differences in early neurological development, gross and fine motor, psychosocial, and facial features of the twins according to Riikonen (1994)

	Twin A	Twin B
Early neurological development		After delivery, jitteriness and irritability for 24 hr
		At 11 months of age, computed tomography (CT) scan of the head showed enlarged cerebral ventricles and moderate cortical and cerebellar atrophy
		EEG study showed moderate universal disturbances, but no epileptiformic episodes.
		Year 1994, MRI showed no significant structural anomalies of the cranium, maturation of the white substance of the brain was retarded
Gross motor		
Stands and walks without support	11 months	
Stands with support		14 months
Walks with support		17 months
Walks without support		2 years
		Increased muscle tone in legs, toe‐standing, truncal hypotonia, ataxia
Fine motor		
Forefinger‐thumb grasping	8 months	Grasps with whole hand at 17 months
Psychosocial		
Social smile	8 weeks	10 months
Monotonic vocalization		10 months
Says two clear words	14 months	
Two different sounds, no babbling or speech imitation		17 months
Plays interactive games	14 months	
Throws toys		17 months
Likes adult to show book, long babbling conversation	17 months	
Two different sounds, no babbling		17 months
Difficult separation of mother		17 months
Facial features	Normal features	The most prominent craniofacial features included right‐sided flat occiput, short palpebral fissures (1.9 cm), thin upper lip, long philtrum (1.5 cm), and maxillary and mandibular hypoplasia
		Facial features remind FAS phenotype
Social situation		
Taken into custody by the social welfare board	3 months	3 months

Toxic effect of alcohol is caused partly by its oxidation product acetaldehyde, which is highly reactive toward DNA, and consequently damages chromosomes and mutates stem cells (Garaycoechea et al., 2018; Wang et al., 2000). Hence it is not surprising that in recent studies, 13%–14% of individuals diagnosed with FASD had chromosomal deletions and duplications that could at least partly explain the FASD‐associated features (Jamuar, Picker, & Stoler, 2018; Zarrei et al., 2018). G‐banding of chromosomes was done for twin B right after the birth to detect potential chromosomal arrangements, but the karyotype was normal, 46, XX. To improve the resolution of the chromosomal analysis, we performed microarray‐based comparative genomic hybridization (aCGH) that allows detecting as small as 50–200 kb copy number variations.

**Figure 1:**
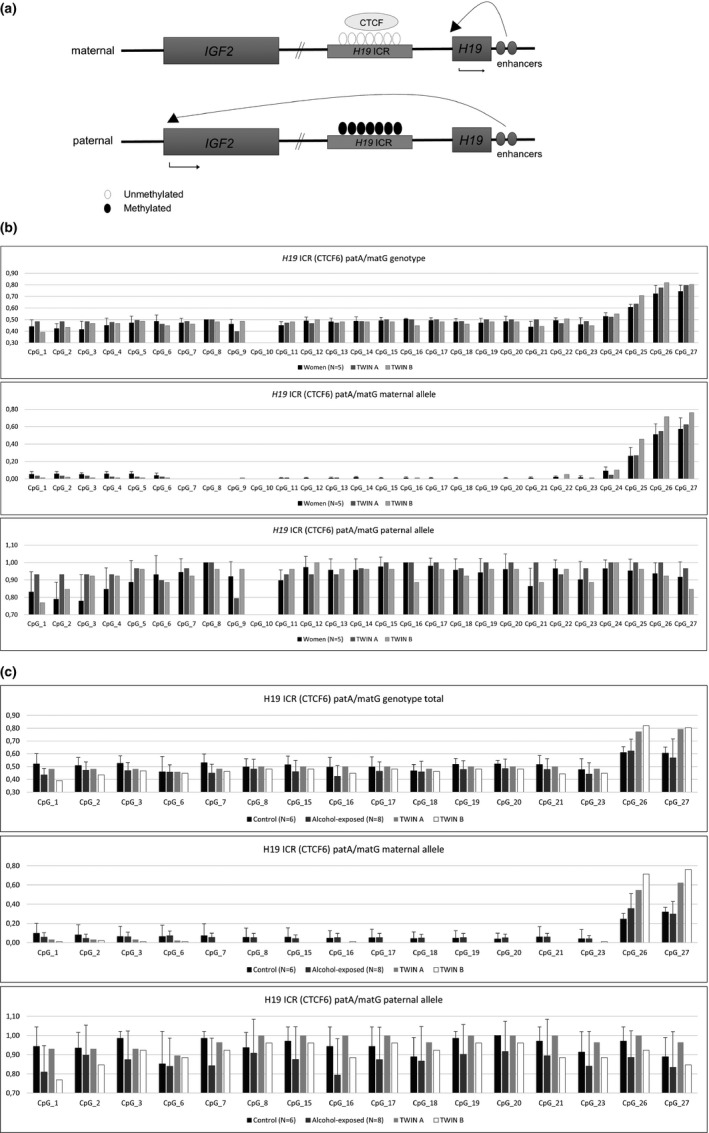
Schematic figure of the insulin‐like growth factor 2 (*IGF2)/H19* locus on chromosome 11p15.5 as well as methylation profiles in both (total) maternal and paternal alleles of imprinting control region at the *IGF2/H19* locus in white blood cells (WBCs) and placental tissue. (a) CTCF protein binds to the maternal (mat) unmethylated imprinting control region (*H19* ICR, NC_000011.10), which blocks the interaction between downstream enhancers and the *IGF2* promoter, and enables the expression of maternal *H19*. The methylation of paternal (pat) *H19* ICR prevents the binding of CTCF protein, allowing access of downstream enhancers to the *IGF2* promoter and provoking the expression of paternal *IGF2*. (b) White blood cells of prenatally alcohol‐exposed twins compared to control women (average methylation level between five women, error bars denote the standard deviation) (c) alcohol‐exposed twins discordant to birth weight compared to control and alcohol‐exposed placentas (average methylation level, error bars denote the standard deviation). Twin B had significantly smaller birth weight compared to twin A

In addition to genetic differences, we were interested in PAE‐induced epigenetic alterations, which have been observed in several previous studies (Kaminen‐Ahola et al., 2010; Marjonen et al., 2017; Portales‐Casamar et al., 2016). In our recent study, we explored the effects of PAE on imprinted *IGF2/H19* locus on chromosome 11p15.5 (Figure 1a). This locus is essential for normal embryonic and placental growth and the two genes are reciprocally imprinted: *IGF2*, a growth promoter, is silenced in maternal allele, whereas *H19*, a negative growth controller, is silenced in paternal allele (DeChiara, Robertson, & Efstratiadis, 1991; Gabory et al., 2009). The *IGF2/H19* imprinting control region regulates the function of the locus by containing binding sequence for CTCF zinc finger regulatory protein (Phillips & Corces, 2009). This sixth CTCF binding site, which contains the observed polymorphism rs10732516 G/A, is normally hypomethylated in maternal and hypermethylated in paternal allele (Figure 1a). Decreased methylation level of this region has been detected often in Silver–Russell syndrome with slow growth (Peñaherrera et al., 2010). Interestingly, when examining methylation level in heterozygous paternal (pat) G/maternal (mat) A and patA/matG placentas, we observed decreased methylation level only in paternal allele of patA/matG genotype in alcohol‐exposed placentas (Marjonen et al., 2017). We also saw an association between the genotype and head circumference (HC) of newborns, which is extremely interesting since the HC has been used in the diagnosis of FASD.

Surprisingly, both of the twins have this particular rs10732516 patA/matG genotype and thus we studied whether alcohol‐induced genotype‐specific decreased methylation level at *IGF2/H19* locus could be detected in their white blood cells (WBCs). Furthermore, we examined the association between the methylation level of this genotype and retarded growth: owing to the restricted growth in FASD phenotype, we explored if similar decreased methylation level could be seen in addition to alcohol‐exposed placentas (Marjonen et al., 2017) also in twin B who had growth‐restricted phenotype at birth.

## 2 MATERIALS AND METHODS

Informed consent was obtained from participants and as a guardian, twin A gave a consent on behalf of twin B. The study was approved by the Ethics Committee of Helsinki University Central Hospital (HUS/1778/2016 and 386/13/03/03/2012).

### 2.1 DNA extraction

White blood cells' DNA of five healthy control women (age 33 ± 5,6 years) was extracted from frozen blood samples using NucleoSpin Blood Kit (Macherey‐Nagel, Düren) according to the manufacturer's protocol. WBCs of twins were extracted from fresh blood samples (described in Marjonen et al., 2018). WBC DNA for molecular karyotyping and methylation analysis was extracted by AllPrep DNA/RNA/miRNA Universal Kit according to the manufacturer's protocol (Qiagen, Valencia). The extraction of placental DNA is described in Marjonen et al. (2017).

### 2.2 Comparative genomic hybridization array

Microarray‐based comparative genomic hybridization was done in the laboratory of Helsinki University Hospital (HUSLAB) using the Agilent Human Genome CGH Microarray Kit 180K (Agilent Technologies Inc.). Data analysis was performed using Agilent Cytogenomics version 4.0.3.12. All nucleotide positions refer to the Human Genome Feb 2009 Assembly (GRCh37/hg19). Those copy number variations (deletions or duplications) that are categorized as clinically irrelevant at the Database of Genomic Variants (http://projects.tcag.ca/variation) were not reported.

### 2.3 Genotyping and *H19* ICR methylation profile analysis by bisulfite sequencing

To find out the genotypes as well as the methylation profile at the CTCF6 at *H19* ICR (NC_000011.10) of twins, we performed bisulfite sequencing. Due to imprinting, the paternal and maternal alleles could be distinguished by sequencing method. Two separate bisulfite conversions were performed for 500 ng of genomic DNA (EZ DNA Methylation™ kit, Zymo Research, Irvine) and pooled afterwards. To avoid possible PCR bias, three independent 20 μl PCR reactions (HotStar PCR kit, Qiagen, Valencia) were performed per sample. Primers were obtained from previous publication to detect the polymorphism (Coolen, Statham, Gardiner‐Garden, & Clark, 2007). PCR reactions were gel isolated, and the three reactions of each sample were pooled and purified using NucleoSpin Gel and PCR Clean‐up Kit (Macherey‐Nagel, Düren). The purified PCR fragments were ligated into pGEM®‐T Easy Vector (Promega, Madison) and cloned by standard protocol. The recombinant DNA clones were purified using NucleoSpin® Plasmid EasyPure kit (Macherey‐Nagel, Düren). On average, 124 clones of both twins were sequenced. The sequences were analyzed by BIQ Analyzer (Bock et al., 2005) excluding the clones with lower than 90% conversion rate from the dataset.

Since twins were patA/matG genotype, bisulfite sequencing was also performed for five patA/matG control women (50–60 clones per woman) for comparison. Moreover, twins were also compared to eight patA/matG alcohol‐exposed and six control placental methylation profiles obtained from our previous publication (Marjonen et al., 2017).

## 3 RESULTS

### 3.1 Molecular karyotyping

Microarray‐based comparative genomic hybridization revealed that twin A had a normal, but twin B an abnormal karyotype arr(GRCh37) 18q12.3‐q21.1 (38,902,336–46,551,876)x1. There was no indication of mosaicism. The deletions of the long arm of chromosome 18 are typically de novo mutations with an incidence of 1 in 40,000 live births (Cody et al., 1999). 18q deletion syndrome (OMIM#601808) is characterized in the literature and the phenotype is highly variable depending on the size, location, and gene content of the deletion. The most common phenotypical features are intellectual disability, short stature, characteristic facial dysmorphism, cleft lip or palate, delayed myelination, foot deformities, and congenital aural atresia (Cody et al., 1999, 2015; Feenstra et al., 2007).

The observed clinically significant 7.65 Mb microdeletion consists of 48 genes, of which 28 genes encode Ensembl gene producing proteins (gene, *OMIM); phosphatidylinositol 3‐kinase catalytic subunit type 3 (*PIK3C3*, *602609), Ras‐like without CAAX 2 (*RIT2*, *609592), synaptotagmin 4 (*SYT4*, *600103), SET binding protein 1 (*SETBP1*, *611060), solute carrier family 14 member 2 (*SLC14A2*, *601611), solute carrier family 14 member 1 (*SLC14A1*, *613868), sialic acid binding Ig‐like lectin 15 (*SIGLEC15*, *618105), ectopic P‐granules autophagy protein 5 homolog (*EPG5*, *615068), proline‐serine‐threonine phosphatase interacting protein 2 (*PSTPIP2*, *616046), ATP synthase F1 subunit alpha (*ATP5F1A*, *164360), HAUS augmin‐like complex subunit 1 (*HAUS1*, *608775), chromosome 18 open reading frame 25 (*C18orf25*), ring finger protein 165 (*RNF165*), lipoxygenase homology domains 1 (*LOXHD1*, *613072), ST8 alpha‐N‐acetyl‐neuraminide alpha‐2,8‐sialyltransferase 5 (*ST8SIA5*, *607162), protein inhibitor of activated STAT 2 (*PIAS2*, *603567), katanin catalytic subunit A1 like 2 (*KATNAL2*, *614697), elongin A3 family member D (*ELOA3D*), elongin A3 family member B (*ELOA3B*), elongin A3 (*ELOA3*), elongin A2 (*ELOA2*, *609522), haloacid dehalogenase‐like hydrolase domain containing 2 (*HDHD2*), immediate early response 3 interacting protein 1 (*IER3IP1*, *609382), SKI family transcriptional corepressor 2 (*SKOR2*, *617138), SMAD family member 2 (*SMAD2*, *601366), zinc finger and BTB domain containing 7C (*ZBTB7C*, *616591), cap binding complex dependent translation initiation factor (*CTIF*, *613178), SMAD family member 7 (*SMAD7*, *602932) and two noncoding RNAs, keratoconus gene 6 (*KC6*), and long intergenic nonprotein coding RNA 907 (*LINC00907*). Mutant forms of seven genes, *SETBP1, ATP5A1, SLC14A1, EPG5, LOXHD1, IER31P1, SMAD7*, are causally associated with human disease. Mutations in *SETBP1* are known to cause autosomal dominant mental retardation (Coe et al., 2014) and its haploinsufficiency is associated with mild neurodevelopmental delay and verbal development delay (Marseglia et al., 2012). *SYT4* deletion may cause epilepsy at the 18q syndrome (Bouquillon et al., 2011).

### 3.2 Development of twins

The current phenotype and developmental features of the 25‐year‐old twin pair are shown in Table 2. Twin A has a normal, active life. The most prominent features of twin B are the absence of speech and moderate mental delay. The brain magnetic resonance imaging (MRI, 1.5 Tesla magnetron, Siemens) done at 3.5 years of age showed frontal cortical and cerebellar atrophy and delayed myelination (Riikonen, Salonen, Partanen, & Verho, 1999). The Wechsler preschool and primary scale of intelligence test (WPPSI‐R) and peabody picture vocabulary test (PPVT) performed at 17 years of age showed universally low capacity (verbal comprehension, performance, and full‐scale IQ < 3.3). Previous research on chromosome 18q deletion phenotypes has shown central nervous system dysmyelination, expressive speech impairment, and intellectual disability of variable degree (Cody et al., 2015; Feenstra et al., 2007; Marseglia et al., 2012; Bouquillon et al., 2011; Linnankivi et al., 2006). Twin B has minor dysmorphic features like thin upper lip, short palpebral fissures, wide nasal bridge, slightly triangular shaped face, absent fingertips, clinodactyly of the fifth digit, and mild syndactyly of second and third digits but no major anomalies. All these minor dysmorphic features are described in previous studies of 18q deletion syndrome (Bouquillon et al., 2011; Cody et al., 2015; Feenstra et al., 2007; Linnankivi et al., 2006; Marseglia et al., 2012). Growth restriction was not seen after neonatal period (Table 2).

**Table 2:** The current developmental and phenotypic features of the twins

	Twin A	Twin B
Motor	Normal developmental milestones	Walking at 2 years, stumbles
Fine motor	Normal	Grapho‐motoric tasks, object assembly limited (level of 3 years of age), clumsy in use of hands
Cognition	Normal	Moderately mentally delayed
Speech	Verbally talented, large vocabulary	No expressive speech, difficulties in verbal concepts
	Neuropsychological testing at 14 years:	Neuropsychological testing at 17 years: WPPSI*,PPVT**: moderately mentally delayed
	difficulties in some abstract concepts, colors, numbers	Performance IQ is low and comprehension is limited to simplified language
Academic skills	Normal school, good ranking at 15 years,	Severe problems in reading,writing, and numbers
	except mathematics	Special education, extended compulsary 11 years schedule
Attention problems	Yes	Yes
Adult life at 25	Qualified as a cook	Occupational therapy, horse‐riding as a hobby
	Independent life	No somatic health problems
	Living with partner	Living in an institution for educationally impaired persons.
Behavior	Social, activities	Impulsive, cheerful, social adaptation abnormal

	Twin A	Twin B
Sex	Female	Female
Height (cm) at 25 years	163	164.1
Weight (kg) at 25 years	61	70.7
OFC	57 (+2SD)	57.5 (+ *2 SD) n*
Dysmorphic features	None	Slightly triangular face, hypertelorism, short palpebral fissures, slight ptosis, wide nasal bridge, thin upper lip mild syndactyly of II‐III toes
Vision	Normal	Strabismus

*Note:* Error OFC: twin B 57.5 (+2SD). PPVT** = peabody picture vocabulary test; WPPSI* = Wechsler preschool and primary scale of intelligence.

### 3.3 DNA methylation at the *IGF2/H19* locus

We next examined potential allele‐specific alterations in DNA methylation at *H19* ICR of *IGF2/H19* locus in white blood cells. We screened 27 CpG sites on CTCF6, a sixth binding site of CTCF zinc finger regulatory protein, using traditional bisulfite sequencing. When we compared methylation profiles of twins to profiles of five control women, we did not observe alcohol‐induced alterations in WBCs (Figure 1b). In addition to alcohol‐induced alterations in DNA methylation, we explored potential association between methylation level and growth. Hence, the average birth weight of alcohol‐exposed newborns is smaller compared to controls and twin B was remarkably smaller at birth compared to twin A; we compared the methylation profile of control and alcohol‐exposed placentas examined in our previous study (Marjonen et al., 2017) and both twins (Figure 1c). Twin B has lower methylation level in majority of CpG sites in paternal allele compared to twin A, similarly as the alcohol‐exposed placentas compared to controls. However, more growth‐discordant twin samples are needed to show association between the methylation level of this specific region in WBCs and growth.

## 4 DISCUSSION

Several twin studies have shown different fetal susceptibility to the effects of alcohol. The mechanism could be connected to the structure of placental blood vessels, unequal placental sharing of monozygotic twins, different rates of organogenesis, or differences in ethanol degradation (Guilliam & Irtenkauf, 1990). However, studies with monozygotic and dizygotic twins suggest that there is also a genetic component with risk and protective alleles in the etiology of discordant phenotypes.

In this study, we examined genetic differences between the dizygotic twins discordant to FAS phenotype. The karyotype of twin B was tested in 1993 by G‐banding with normal results. We performed aCGH for both twins to gain higher resolution, and we observed a clinically significant 18q12.3‐q21.1 microdeletion in severely affected twin B. This is a plausible explanation for notable, permanent differences in the development and phenotype of twins. The phenotype of 18q deletion syndrome is highly variable (Feenstra et al., 2007; Linnankivi et al., 2006) and overlaps with FASD or FAS phenotype with neuronal disorders, growth restriction, and birth defects. Hence it is not possible to determine the separate effects of PAE and the microdeletion on twin B phenotype.

The microdeletion can be a consequence of heavy maternal alcohol consumption. Alcohol exposure, more specifically acetaldehyde, can damage chromosomes in oocytes or in early embryos before pregnancy have been noticed. We did not observe mosaicism of the microdeletion in leukocytes, which suggests that de novo mutation could have been occurred already in gametes. However, without parental DNA samples and other tissue samples of affected twin, the origin of the mutation and the potential cell type‐specific mosaicism remain vague. Increased incidence of chromosomal arrangements has been found in FASD individuals (Douzgou et al., 2012; Zarrei et al., 2018). Interestingly, one of 12 children diagnosed with FASD by Zarrei et al. (2018) had a duplication in chromosome 18q12.1. This increased incidence of abnormal chromosomal structures might be explained also by DNA hypomethylation (Robertson, 2005). It has been suggested that alcohol consumption decreases the amount of folate, which is needed for cells’ methionine cycle (Halsted et al., 1996). Thus, alcohol could reduce the production of methyl groups for DNA and histone methylation, cause hypomethylation, and consequently decrease the stability of the chromosomes.

Recently, we have observed genotype‐specific decreased DNA methylation level in *IGF2/H19* locus in both alcohol‐exposed and in vitro fertilized (IVF)‐derived placentas, which suggest differences in sensitivity to the effects of environmental factors between the genotypes (Marjonen et al., 2018, 2017). Both of the twins have this specific rs10732516 patA/matG genotype where decreased methylation level was detected, and we examined if these PAE‐associated changes could be seen also in their WBCs. Those alterations could be used as biomarkers in the diagnostics of alcohol‐induced disorders. We explored the effects of PAE on WBCs of twins and five healthy women as controls, but we did not observe associations between PAE and methylation level. This is in line with our previous study, in which IVF treatment was a prenatal environmental factor affecting birth weight. We observed genotype‐specific association between IVF treatment and decreased DNA methylation in this region in placenta, but the difference was not seen in WBCs of newborns (Marjonen et al., 2018). Furthermore, in the previous IVF studies without genotype‐specific approach, hypomethylation in placentas, buccal cells and mononuclear cells of cord blood have been observed, but not in WBCs (Castillo‐Fernandez et al., 2017; Loke, Galati, Saffery, & Craig, 2015; Nelissen et al., 2013). This could be explained by the diverse methylation profiles of cell types in WBC samples.

Owing to the essential role of *IGF2/H19* locus in embryonic and placental growth, we wanted to see if studied twins are discordant in both birth size and methylation level. Interestingly, twin B who had significantly smaller birth weight had lower methylation level in majority of CpG sites compared to twin A who had normal birth weight. We saw this similar trend when we compared alcohol‐exposed placentas to controls in our previous study by Marjonen et al. (2017). Due to the known association between PAE and retarded growth as well as this observed association between PAE and decreased placental DNA methylation, decreased methylation in WBCs of twin B could indicate severely retarded growth. Although the direction of methylation changes in this regulatory region is similar and could be associated with retarded growth in both alcohol‐exposed placentas and twin B with deletion, alteration in WBCs is subtle and should be confirmed with larger number of samples.

Due to the highly variable phenotype of FASD and the complex interaction between genome, epigenome, and environment, the molecular mechanisms causing alcohol‐induced developmental disorders are challenging to reveal. Contribution of genetic factors to the FASD phenotype can be a combination of chromosomal abnormalities, risk/protective alleles, and/or epigenetic changes. In attempts to clarify the role of genetics in FASD phenotype, it is pivotal to separate these factors from each other and therefore, children suspected to have FASD should be tested by high‐resolution method, like aCGH, to exclude identify chromosomal etiologies.

## CONFLICT OF INTEREST

No competing interests are declared.

## Data Availability

The data that support the findings of this study are available from the corresponding author upon reasonable request.
